# Structural and functional thalamocortical connectivity study in female fibromyalgia

**DOI:** 10.1038/s41598-021-02616-1

**Published:** 2021-12-02

**Authors:** Dajung J. Kim, Manyoel Lim, June Sic Kim, Chun Kee Chung

**Affiliations:** 1 Department of Brain and Cognitive Sciences, Seoul National University College of Natural Sciences, Seoul, 08826 Republic of Korea; 2 Neuroscience Research Institute, Seoul National University College of Medicine, Seoul, 08826 Republic of Korea; 3 Research Institute of Basic Sciences, Seoul National University, Seoul, 08826 Republic of Korea; 4 Department of Neurosurgery, Seoul National University Hospital, 101 Daehak-ro, Jongno-gu, Seoul, 03080 Republic of Korea; 5 Present Address: Department of Biologic and Materials Sciences and Prosthodontics, University of Michigan School of Dentistry, Ann Arbor, MI 48109 USA

**Keywords:** Chronic pain, Pain, Thalamus

## Abstract

Dysfunctional thalamocortical interactions have been suggested as putative mechanisms of ineffective pain modulation and also suggested as possible pathophysiology of fibromyalgia (FM). However, it remains unclear which specific thalamocortical networks are altered and whether it is related to abnormal pain perception in people with FM. Here, we conducted combined vertex-wise subcortical shape, cortical thickness, structural covariance, and resting-state functional connectivity analyses to address these questions. FM group exhibited a regional shape deflation of the left posterior thalamus encompassing the ventral posterior lateral and pulvinar nuclei. The structural covariance analysis showed that the extent of regional deflation of the left posterior thalamus was negatively covaried with the left inferior parietal cortical thickness in the FM group, whereas those two regions were positively covaried in the healthy controls. In functional connectivity analysis with the left posterior thalamus as a seed, FM group had less connectivity with the periaqueductal gray compared with healthy controls, but enhanced connectivity between the posterior thalamus and bilateral inferior parietal regions, associated with a lower electrical pain threshold at the hand dorsum (pain-free point). Overall, our findings showed the structural thalamic alteration interacts with the cortical regions in a functionally maladaptive direction, leading the FM brain more responsive to external stimuli and potentially contributing to pain amplification.

## Introduction

Fibromyalgia (FM) is one of the most common and devastating chronic pain disorders, affecting approximately 10–12% of the population with twice higher prevalence among women^1^. FM exhibits widespread musculoskeletal pain accompanied by low sleep quality, cognitive impairments, and emotional dysregulation^2^. While the FM’s etiology remains elusive, central sensitization, including enhanced facilitation or compromised descending pain modulation, has been suggested as the primary contributor to widespread pain symptoms^3–5^.

Over the past 20 years, a growing amount of evidence has shown structural^6–8^, functional^9–14^ and neurochemical^15–17^ brain alterations in FM supporting nociplastic pain features contributing to clinical symptoms^5,18^. Of note, it has been suggested that a disrupted thalamo-cortical (TC) network contributes to impaired pain modulation manifested in FM. In an earlier fMRI experiment, individuals with FM exhibited less activation in the pulvinar nucleus of the thalamus and pain-modulatory regions such as rostral anterior cingulate cortex (rACC) when pressure pain, comparable intensity as controls, was administered to individuals with FM^19^. They further showed that individuals with FM exhibited less functional connectivity between the thalamus and orbitofrontal cortex in the same experimental setting^20^.

Moreover, individuals with FM appeared to have disrupted the resting-state TC network system. Flodin et al. (2014) observed that increased pain sensitivity representing hyperalgesia was associated with increased left thalamus-prefrontal cortex connectivity^21^. We also reported that individuals with FM had abnormal spontaneous oscillatory activity in the sensory, motor, and prefrontal cortex, associated with affective qualities of pain^22^, further supporting the idea that the TC network might underlie individual differences in pain perception.

It is noteworthy that each thalamic nucleus exerts its role in cognition, emotion, and pain by interacting with the preferentially connected cortex^23–26^. However, less is known about which specific TC networks, either structurally or functionally, are abnormal in people with FM. It has been suggested that the thalamus plays an active role by regulating brain signals transmitted to cortical areas^24^. The higher-order thalamus, such as the pulvinar, even regulates cortico-cortical information transmission^27^. Accordingly, we assumed that structural or functional TC networks seeded from regional pathology of the thalamus would capture abnormal TC relationships presented in people with FM.

Thus, our study goals were to (1) find a regional alternation of the thalamus, (2) map structural and functional interaction of the specific thalamus with cortical areas, and (3) relate TC functional connectivity and experimental pain sensitivity for pain-free body area. To this end, we applied multiple analytical methods, including vertex-wise subcortical shape, cortical thickness, and structural covariance (SC), which correlates morphological values between brain regions and resting-state functional connectivity. We expected that this approach would provide complementary evidence regarding the contribution of the TC network to elevated pain perception in FM.

We hypothesized that the FM group would exhibit structural thalamic atrophy in the posterior subnuclei based on previous structural (white and gray matter morphology) and functional connectivity studies^28–30^. The expected thalamic atrophy would have, in turn, an abnormal structural and functional relationship with posterior parietal cortical regions, in particular, considering its preferential connection as revealed by diffusion tensor and functional neuroimaging studies^31–33^. Lastly, we tested whether functional connectivity of the TC networks, in which individuals with FM exhibited abnormal patterns, are associated with experimental pain sensitivity (pressure and electrical pain threshold) of FM.

## Methods

### Participants

Nineteen right-handed female primary FM patients according to the American College of Rheumatology 1990 criteria for the classification of FM^34^ were recruited from rheumatology departments at Seoul National University Hospital (SNUH) (n = 9) and Hallym University Sacred Heart Hospital (n = 10) through local advertisements. All experiments except the diagnosis were conducted at the SNUH to ensure the reliability of the results. Eligibility criteria for individuals with FM were: (1) between 30 and 60 years of age, (2) having a duration of widespread pain at least 3 months, (3) reporting an average pain intensity of at least 40 on the 0- to 100-mm pain visual analog scale (VAS) over the past week, and (4) willing to stop taking medications known to influence the somatosensory system (eg, analgesics, antidepressants, and anticonvulsants) at least 3 days before the assessments. Participants were excluded if they had: (1) secondary FM associated with inflammatory arthritis, (2) a medical history of psychiatric disorders (i.e., major depressive disorder, schizophrenia, substance abuse) or other disorders affecting the central nervous system (i.e., cerebrovascular accident, multiple sclerosis, Parkinson’s disease), (3) signs of peripheral neuropathy at the upper extremities (e.g., related to trauma, polyneuropathy), (4) concomitant acute pain in the upper extremities (e.g., due to injury), (5) pregnancy, or (6) contradictions for magnetic resonance imaging (MRI) (i.e., dental braces, permanent retainers, pacemakers, surgical clips, metallic implants) assessments. Age-, sex-, and education level matched pain-free healthy controls (HCs) (n = 21) were also enrolled in the study. Exclusion criteria were same as for FM. None of the subjects reported the presence of pain in the hand. The study protocol was approved by the Institutional Review Boards at Seoul National University Hospital (H-1107-013-367) and Hallym University Sacred Heart Hospital (2011-I048) and performed under the Helsinki guideline for research involving humans. All participants were informed about the study procedures and gave written informed consent before study participation.

### Clinical questionnaires and quantitative sensory testing

All participants were asked to complete basic demographic and clinical questionnaires, including mood [Beck Depression Inventory (BDI)], clinical pain [VAS, short-form McGill Pain Questionnaire (SF-MPQ)]. Quantitative sensory testing data in the current study were previously published elsewhere^12^, showing that FM group exhibited a significantly lower pressure (PPT) and electrical pain threshold (EPT). The PPT was measured by applying mechanical stimuli on the hand thenar eminence (neutral point) with an experimenter-operated pressure algometer (Baseline Evaluation Instruments, Fabrication Enterprises, New York, USA). Specifically, an in-house Teflon stimulation surface of 1 cm^2^ and a slope of 0.5 kg per second were used. While the researcher was increasing pressure level, the subjects were asked to indicate verbally when they first noticed the stimulus as painful. Also, we measured the EPT on the hand dorsum (neutral point) using an intra-epidermal electrode (Nihon-Kohden, Tokyo, Japan) which can selectively stimulate cutaneous Aδ nociceptors^35^. The EPT was determined by increasing the stimulator current (pulse width: 1 ms) output in steps of 0.3 mA (Digitimer DS7AH, Digitimer Ltd., United Kingdom). The subjects were asked to indicate verbally when they first perceived the stimulus as painful. All experimental pain threshold tests were performed on the right (dominant) hand. Each PPT and EPT was determined 3 times, and averaged values were used for further analysis. The individual pain threshold was included as an indicator of experimental pain sensitivity for the FM group. Demographic and clinical variables were compared between the two groups by independent t-test and chi-square test for continuous and categorical values, respectively.

### MRI acquisition

All MRI data were collected with a Magnetom TrioTim 3T scanner (Siemens, Erlangen, Germany) at the Seoul Nation University. High-resolution T1-weighted images were scanned from all participants except 1 HC due to claustrophobia with the following parameters: repetition time [TR] = 1670 ms, echo time [TE] = 1.89 ms, flip angle = 9°, field of view [FOV] = 250 mm, a sagittal acquisition with 256 × 256 pixel matrix, voxel size = 1 × 1 × 1 mm, slice thickness = 1.0 mm with no gap; excitation = 1. To screen for mass lesions or pathological hyper-intensities of the brain, T2-weighted images were also acquired with the following parameters: [TR] = 5160 ms, [TE] = 91 ms, flip angle = 131°, FOV = 220 mm, an axial acquisition with 640 × 580 pixel matrix, slice thickness = 5.0 mm. Thus, a total of 19 FM and 20 HC subjects were included in the structural MRI analyses.

Resting-state functional MRI (fMRI) data were acquired using echo‐planar imaging sequence: repetition time = 3500 ms; echo time = 30 ms; flip angle = 90°; FOV = 240 mm; voxel size = 1.9 × 1.9 × 3.5 mm; slice thickness = 3.5 mm; total 116 volumes. The participants were instructed to keep their eyes closed, stay awake, and relax during the resting-state fMRI scan. They were asked to keep their head as still as possible during the scan. Head motion was minimized by the placement of foam padding around the head. We did not acquire fMRI data from the initial 7 FM subjects. Thus, a total of 12 FM and 20 HC subjects were included in the functional MRI analysis.

### Thalamic volume and shape analysis

Automatic segmentation of the left and right thalamus from T1-weighted images and subsequent vertex-based thalamic shape analysis to detect the location of regional change of the thalamus was performed using FSL's FMRIB Integrated Registration and Segmentation Tool (http://fsl.fmrib.ox.ac.uk/fsl/fslwiki/first)^36^. Thalamic segmentation is based on the shape/appearance models constructed from manually segmented images of 336 training data^37^. We visually inspected the accuracy of segmentation results and confirmed that all segmented thalami were eligible for further analysis. Parameterized surface mesh for each thalamus was done in a way that the numbers of vertices, an apex of adjoining triangles of the mesh are fixed to every subject. Each mesh was then aligned to the average mesh of a training dataset while removing translation and rotation factors by minimizing the sum-of-squares difference of each subject's corresponding vertices and the average shape in the Montreal Neurological Institute (MNI) 152 space. Then, the vertex location of each subject was projected to the average shape of the surface normal.

The group difference of the thalamic volume was assessed by analysis of covariance (ANCOVA) while age and intracranial volume were included as covariates of no interest. We estimated each subject’s intracranial volume by using Freesurfer v5.3.0 (http://surfer.nmr.mgh.harvard.edu) to control brain size. Also, the group differences of vertex measures of each right or left thalamus were carried out using F-statistics while controlling for age. Multiple comparisons were corrected with the threshold-free cluster enhancement method (family-wise error (FWE) of *p* < 0.05) using the ‘randomize’ function in FSL. And then, a thalamic mask from the region showing a significant group difference was generated. The resulting significant region was overlaid on the high-resolution 3-dimensional atlas of the human thalamic structures generated from multiple histologic data in MNI space^38^ and reconstructed by using 3D slicer (https://www.slicer.org) software to show anatomically specific thalamic alteration of the FM group. An averaged scalar value, which represents regional change compared to the average normal surface, within the mask was extracted from each subject using the ‘fslstats’ function in FSL.

### Cortical thickness analysis

The Constrained Laplacian Anatomic Segmentation using proximity (CLASP) surface extraction procedure was applied to measure the cortical thickness^39^. Briefly, cortical thickness was calculated in individuals’ native space as the distance between white and gray interfaces at 40,962 vertices in each hemisphere and registered to the MNI ICBM152 template to facilitate a group comparison. Thickness data was smoothed using 20-mm full-width at half-maximum Gaussian kernel to reduce measurement noise while preserving cortical topological features^40^. Group differences of cortical thickness at each vertex while controlling the effect of age and average thickness were tested by the general linear model (GLM) using the SurfStat toolbox (http://www.math.mcgill.ca/keith/surfstat/) in Matlab (R2016b, MathWorks, Natick, MA, USA). Multiple comparisons were corrected based on the Random Field Theory (RFT) (RFT-corrected *p* < 0.05). The RFT controls the probability of ever finding a false positive. The resulting vertex-wise maps for group difference of cortical thickness were visualized by using the SurfStat toolbox (http://www.math.mcgill.ca/keith/surfstat).

### Thalamo-cortical structural covariance mapping

First, to map the TC-SC, we assessed the GLM model, including the vertex-wise thickness of the all cortical surface as a dependent variable, scalar projection values of the thalamus as independent variables, and age and average thickness as covariates. Next, to localize abnormalities in the FM group's TC-SC, we assessed GLM, including the vertex-wise thickness of the all cortical surface as a dependent variable, diagnosis, scalar projection values of the thalamus, and interaction between them as independent variables, and age and average thickness as covariates. The resulting vertex-wise maps for group difference of SC were visualized by using the SurfStat toolbox.

### Seed-based functional connectivity

The preprocessing steps adapted from the 1000 Functional Connectomes Project (http://www.nitrc.org/projects/fcon_1000) were performed with the FSL (http://www.fmrib.ox.ac.uk/fsl) and AFNI (http://afni.nimh.nih.gov/afni) softwares. After discarding the first four volumes, slice time correction, motion correction, grand-mean scaling, removing of nuisance signals (cerebrospinal fluid, white matter, and six motion parameters) by regression, removing of linear and quadratic trends, spatial smoothing using a Gaussian kernel of 6 mm full-width half-maximum (FWHM), and temporal band-pass filtering (0.01–0.08 Hz) were performed. The preprocessed images were then transformed to the MNI 152 (2 mm) template.

We created a spherical seed (6-mm radius) in the posterior thalamic region showing between-group differences (FM vs. HC) in shape analysis. The average time series of seed region was extracted in standard space and then correlated with time course from all other voxels in the brain in native space to create an intrinsic functional connectivity map. The resulting functional connectivity maps were Fisher’s r to z transformed and then transformed into standard space. We performed a voxel-wise group comparison between FM and HC groups using an independent sample *t*-test. Age was included as a covariate of no interest. The significance threshold was set to voxel-level *Z* > 3 (uncorrected), combined with a cluster-extent threshold of *p* < 0.05 (FWE-corrected). The results were visualized using BrainNet Viewer^41^ and DPABI Viewer^42^.

### ROI-to-ROI functional connectivity analysis

We further performed posterior thalamus-to-region of interest (ROI) functional connectivity analysis to test specific hypotheses regarding the interaction between the thalamus and descending pain modulatory circuit and hypothalamus. Spherical ROIs with the 6-mm radius were defined for right rACC (x = 8, y = 46, z = 4)^19^ and PAG (x = 0, y = − 32, z = − 10)^43,44^. Spherical ROIs with the 2-mm radius were defined for bilateral medial (x =  ± 4, y = − 2, z = − 12) and lateral hypothalamus (x = ± 6, y = − 2, z = − 12)^45^ (Fig. 5A). The ROIs were linearly transformed to each subject’s functional space, and the mean time series (0.01–0.08 Hz) across all voxels in the ROIs were extracted. Temporal correlation between thalamus and each of these regions was calculated and then transformed into Z values using Fisher's r to Z transformation. The group difference of functional connectivity was assessed by ANCOVA while age was included as covariates of no interest. The Benjamini–Hochberg false discovery rate correction (*q* = 0.05)^46^ was applied for correcting multiple comparisons functional connectivity.

### Clinical significance of cortical thickness and thalamo-cortical functional connectivity

Associations between the pain threshold (PPT and EPT) and cortical thickness in cluster of group differences were examined by using Pearson’s correlation coefficient. The significance threshold was set at *p* < 0.025 (0.05/2, to account for two different pain thresholds). We performed whole-brain voxel-wise regression analysis with the pain threshold as a regressor of interest to examine correlations between functional connectivity and pain thresholds, including PPT and EPT. The significance threshold was set to voxel-level *Z* > 3 (uncorrected), combined with a cluster-level FWE-corrected *p* < 0.025 (0.05/2, to account for two different pain thresholds). We examined the potential association between altered thalamic shape displacement, cortical thickness, TC functional connectivity shown in the FM group and BDI to see if any of these changes were related to depressive symptoms using Pearson's correlation. *p* < 0.05 was taken as a prior significant threshold.

### Head motion analysis

We calculated the frame-wise displacement (FD), an index of head motion^47^, for each subject. There was no significant group difference in mean FD (FM: 0.077 ± 0.04; HC: 0.067 ± 0.017, *p* = 0.36).

## Results

There was no significant difference in demographic characteristics between the groups except pain threshold and mood symptoms. Detailed information is listed in Table 1.

**Table 1:** Demographic and clinical characteristics of study participants.

	Fibromyalgia (n = 19)	Healthy controls (n = 20)	P value
Female, no. (%)	19 (100)	20 (100)	–
Age at assessment, y	44.9 (8.3)	45.0 (8.4)	0.999
Education > 12 y, no. (%)	8 (42.1)	7 (35.0)	0.648
Pain duration, months	35.6 (31.1)	–	–
Tender point, no. (0–18)	15.7 (1.8)	1.5 (2.2)	< 0.001
Beck Depression Inventory Score	19.0 (6.8)	3.0 (4.0)	< 0.001
**Medication, no. of patients (%)**^**a**^			
Analgesics/muscle relaxants/NSAIDs	14 (74%)	–	–
Antidepressants	14 (74%)	–	–
Anticonvulsant	7 (37%)	–	–
Pain intensity VAS—last week	52.8 (20.3)	–	–
Pressure pain threshold at thenar eminence (kg)	2.85 (0.8)	3.75 (0.6)	< 0.001
Electrical pain threshold at hand dorsum (mA)	1.02 (0.35)	1.49 (0.69)	0.013

Data are presented as mean ± SD or number of subjects (%).

FIQ, Fibromyalgia Impact Questionnaire; NSAIDs, nonsteroidal anti-inflammatory drugs; VAS, visual analog scale.

^a^The numbers are not mutually exclusive.

### Thalamus morphometry and cortical thickness using MRI

**Figure 1:**
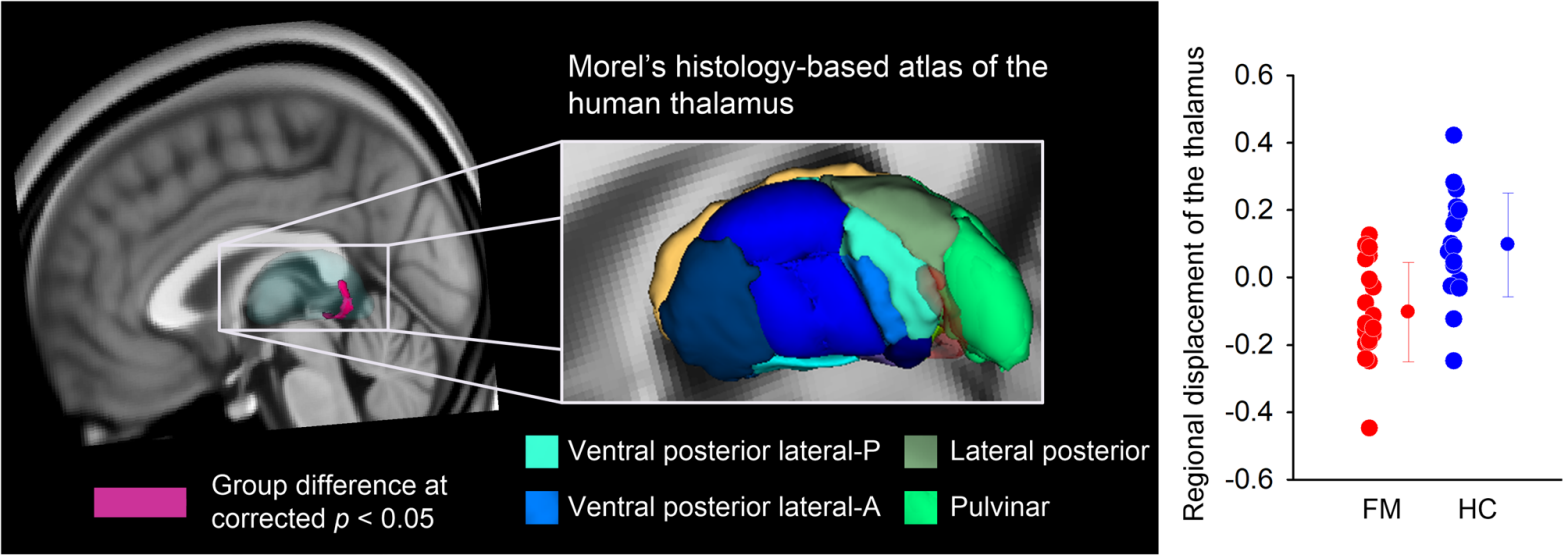
Regional thalamic atrophy in individuals with fibromyalgia (FM) by surface-based shape analysis. The thalamic surface area where FM showed hypotrophy, inward deformation from the mean thalamic surface, compared with healthy controls (HC) was colored as pink (corrected *p* < 0.05). Significant results are also overlaid on Morel's histology-based atlas^38^ reconstructed for visualization purposes. Red dots represent FM (n = 19), and blue dots represent HC subjects (n = 20). Line graphs are expressed as mean ± standard deviation. P, posterior; A, anterior.

The volumetric analysis showed that mean thalamic volumes were 7834.2 ± 616.7 mm^3^ on the left and 7624.0 ± 582 mm^3^ on the right in the individuals with FM, and 8038.1 ± 715.6 mm^3^ on the left and 7828.9 ± 684.7 mm^3^ on the right in the HCs. The FM group had numerically lower thalamic volume; however, there were no significant differences between the two groups in both the left (*f*(1,35) = 0.43, *p* = 0.52) and right thalamic volume (*f*(1,35) = 0.56, *p* = 0.46). Nonetheless, vertex-wise shape analysis showed that the FM group exhibited inward deformation along the posterior areas encompassing pulvinar and VPL nuclei of the left thalamus compared to the HC group (108 voxels; peak coordinate xyz = − 14, − 28, 0) at corrected *p* < 0.05 (Fig. 1).

**Figure 2:**
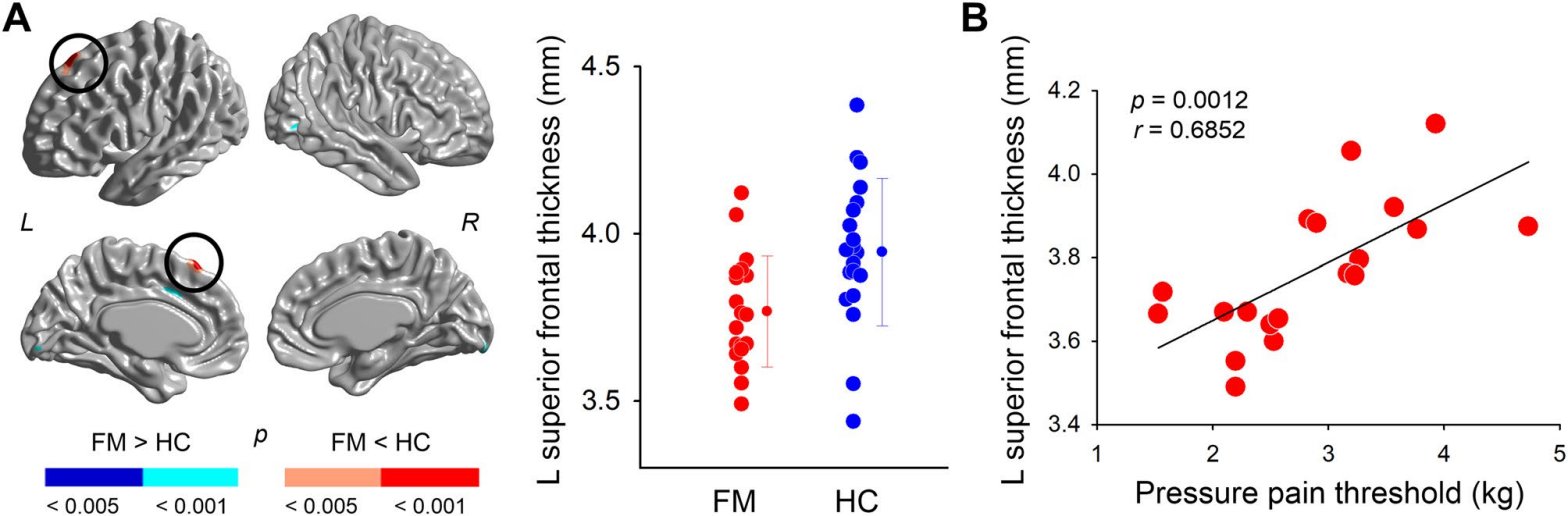
Areas of thinner cortical thickness in individuals with fibromyalgia (FM) than in healthy controls (HC). (**A**) Individuals with FM had less thickness in the left superior frontal cortex (Random field theory-based correction *p* < 0.05). There were no regions where FM had greater thickness than HC. Red dots represent FM (n = 19), and blue dots represent HC subjects (n = 20). Line graphs are expressed as mean ± standard deviation. (**B**) Relationship between thinner cortical thickness and pressure pain threshold over the thenar muscle in patients with FM.

In addition, individuals with FM had thinner cortical thickness in the left superior frontal cortex while controlling for age and average thickness (peak *t* = 5.28; xyz = − 10, 32, 58) at RFT corrected *p* < 0.05 (Fig. 2A). Figure 2B shows the relationship between left superior frontal cortical thickness and PPT in the FM group. FM subjects with thinner superior frontal cortical thickness were more likely to have lower pressure pain thresholds of thenar muscle (r = 0.69, *p* = 0.0012). This relationship was not seen in HC subjects (r = 0.40, *p* = 0.083). There were no other significant correlations with EPT (*p* > 0.05).

### Thalamo-cortical structural covariance analysis using MRI

**Figure 3:**
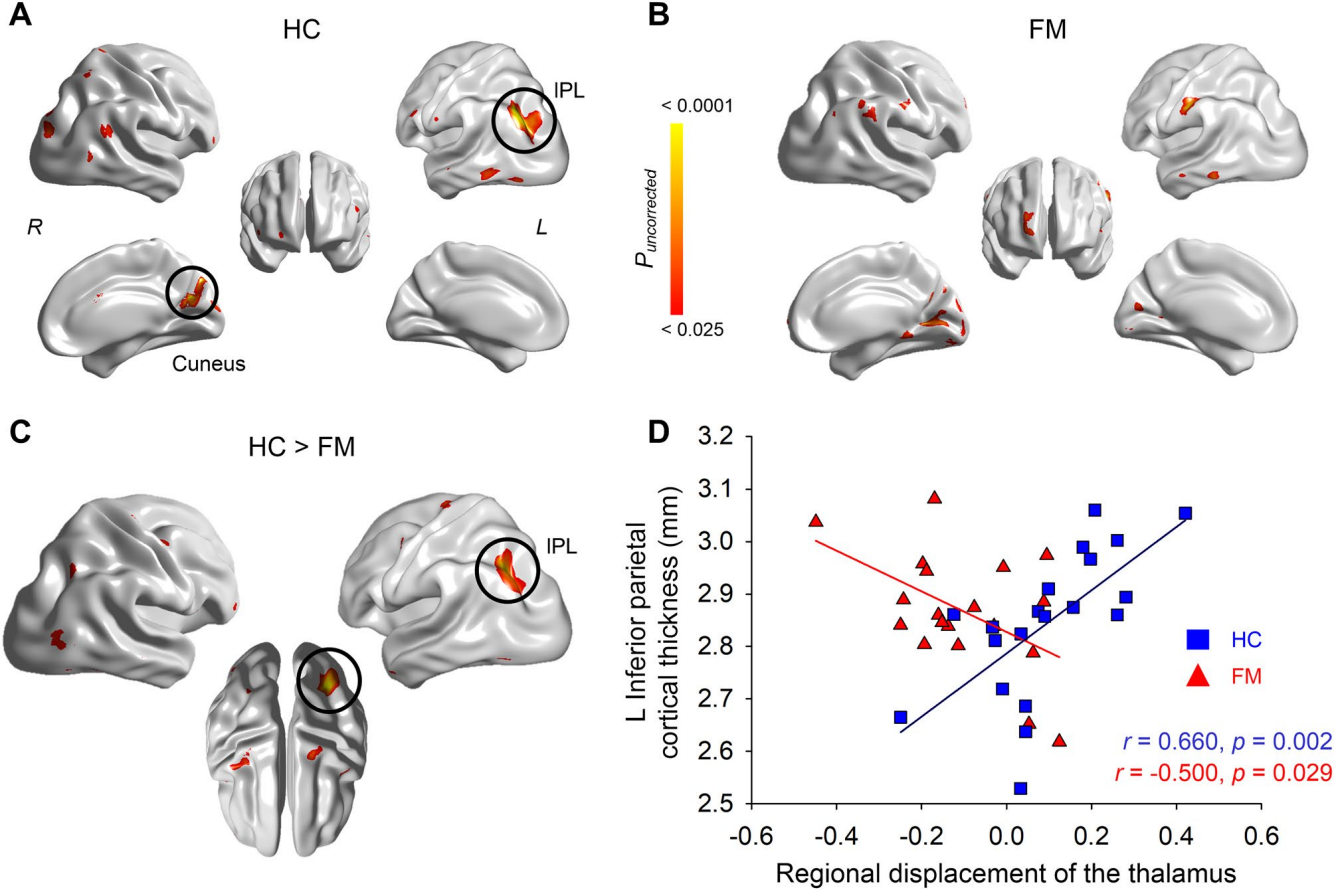
Thalamo-cortical structural covariance of each group and between-group differences. Structural covariance between the seed (pulvinar/VPL nuclei) and the entire surface of (**A**) healthy control (HC) and (**B**) fibromyalgia (FM). (**C**) The interaction effect between seed and diagnostic group on the thickness. For visualization purposes, results with uncorrected trends (*p* < 0.025) were illustrated. Each test was Random-field theory corrected *p* < 0.05, outlined in a black circle. (**D**) Thickness values of the region showing a significant interaction were extracted and correlated with the regional displacement value of the seed (pulvinar/VPL nuclei). VPL, ventroposterior lateral; IPL, inferior parietal lobule.

In a within-group analysis of TC covariance, we found that regional change of the posterior thalamus was strongly covaried with the cortical thickness of the left inferior parietal lobule (IPL) and right cuneus areas in the HC group (Fig. 3A). Conversely, in the FM group, the pattern presented in the HC group was mostly absent. Instead, the posterior thalamus was covaried with cortical thickness in the left postcentral sulcus/supramarginal and right cuneus areas with only a marginal significance (Fig. 3B). The between-group difference assessed by the effect of the interaction term (degree of thalamic regional change * diagnosis) on the thickness revealed that FM group exhibited inversed TC relationship compared to controls in the left IPL at corrected* p* < 0.05 (Fig. 3C, D).

### Seed-based thalamo-cortical functional connectivity using resting-state fMRI

**Figure 4:**
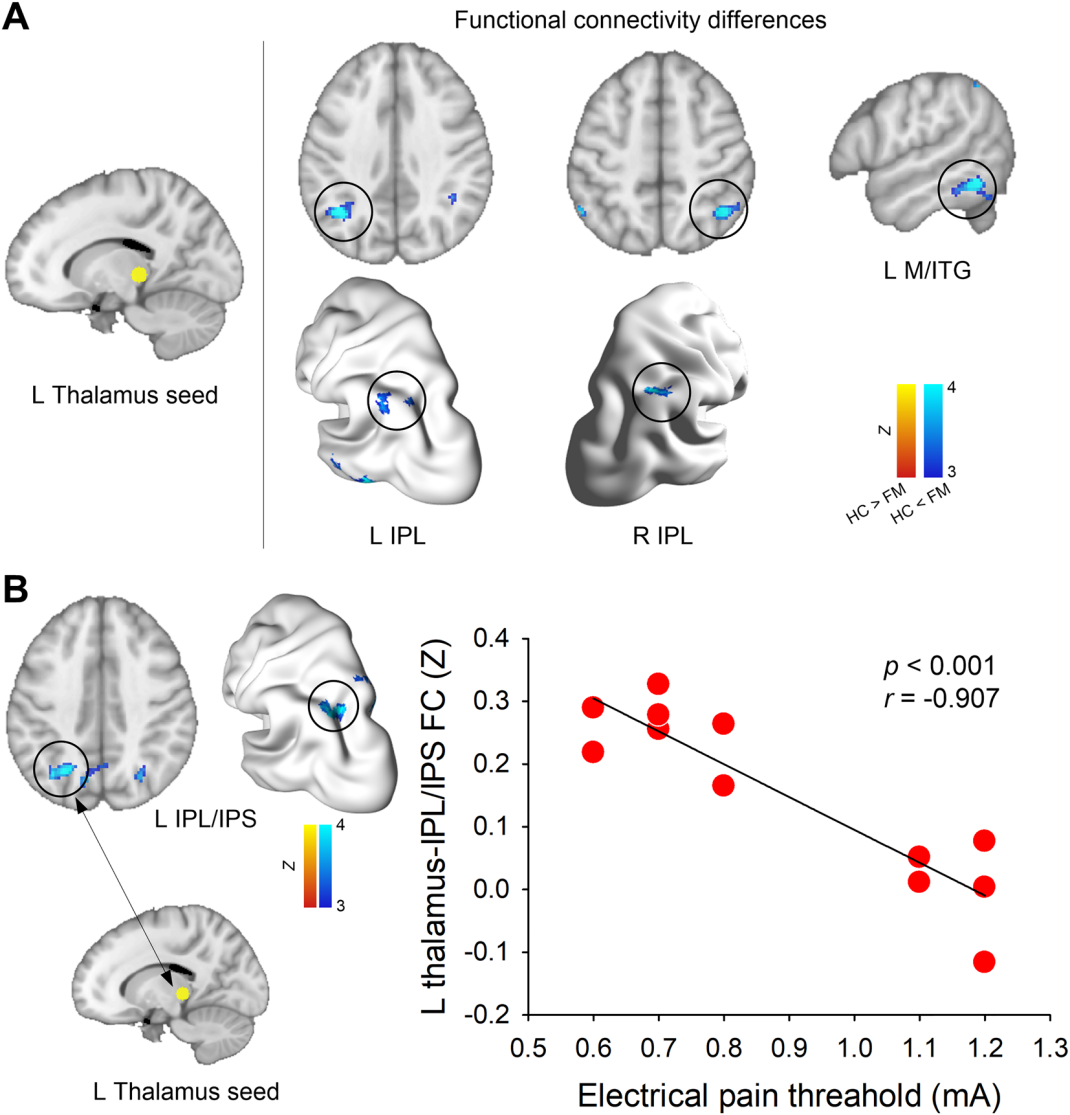
Thalamo-cortical functional connectivity (0.01–0.08 Hz). (**A**) Between-group differences in posterior thalamus functional connectivity. We created a spherical seed (6-mm radius, yellow circle) in the brain region, showing between-group differences (FM vs. HC) in thalamus shape analysis. All statistical images are displayed with significant clusters (voxel-level threshold *Z* > 3 and cluster-level extent threshold *p* < 0.05, FWE-corrected). (**B**) Correlation between posterior thalamus functional connectivity (Z) and electrical pain threshold (mA) on the hand in FM (n = 12). Results are displayed with significant clusters (voxel-level threshold *Z* > 3 and cluster-level extent threshold *p* < 0.025, FWE-corrected). IPL, inferior parietal lobule; M/ITG, middle and inferior temporal gyrus; FC, functional connectivity.

Seed-based functional connectivity analysis revealed that FM group showed increased functional connectivity between the left posterior thalamus (pulvinar) and left/right IPL, including angular gyrus and left middle/inferior temporal gyrus (*p* < 0.05, FWE-corrected) (Fig. 4A) (Table 2). In the FM group, there was a significant negative relationship between EPT and posterior thalamus functional connectivity with IPL, precuneus, and middle cingulate cortex (*p* < 0.025, FWE-corrected) (Fig. 4B) (Table 3). Namely, individuals with FM with increased thalamo-IPL functional connectivity had a lower pain threshold in response to noxious electrical stimulation (*r* = − 0.907). We did not find any significantly correlated regions with EPT in the HC group. Also, there were no other significant correlations with PPT in both groups.

**Table 2:** Between group differences in seed-based functional connectivity.

Seed region	Contrast	Brain region	Peak coordinates (MNI)			Cluster size (number of voxels)	T-score
			x	y	z		
Thalamus	FM > HC	Left inferior parietal lobule	− 40	− 56	36	120	5.5
		Right inferior parietal lobule	38	− 52	54	156	4.8
		Left middle/inferior temporal gyrus	− 54	− 52	− 12	187	5.1
	FM < HC	n.s	–	–	–	–	–

All statistical results were thresholded at voxel-level *Z* > 3 and cluster-level *p* < 0.05, FWE-corrected.

FM, fibromyalgia; HC, healthy control; n.s., not significant; FWE, family-wise error.

**Table 3:** Correlation between thalamus functional connectivity and pain threshold in fibromyalgia.

Variables	Brain region	Peak coordinates (MNI)			Cluster size (number of voxels)	R value
		x	y	z		
Electrical pain threshold	Left inferior parietal lobule/sulcus	− 36	− 66	42	244	− 0.92
	Left precuneus	− 12	− 66	46	317	− 0.93
	Right middle cingulate cortex	10	− 6	44	137	− 0.90
Pressure pain threshold	n.s	–	–	–	–	–

All statistical results were thresholded at voxel-level *Z* > 3 and cluster-level *p* < 0.025, FWE-corrected.

### ROI-to-ROI functional connectivity analysis

**Figure 5:**
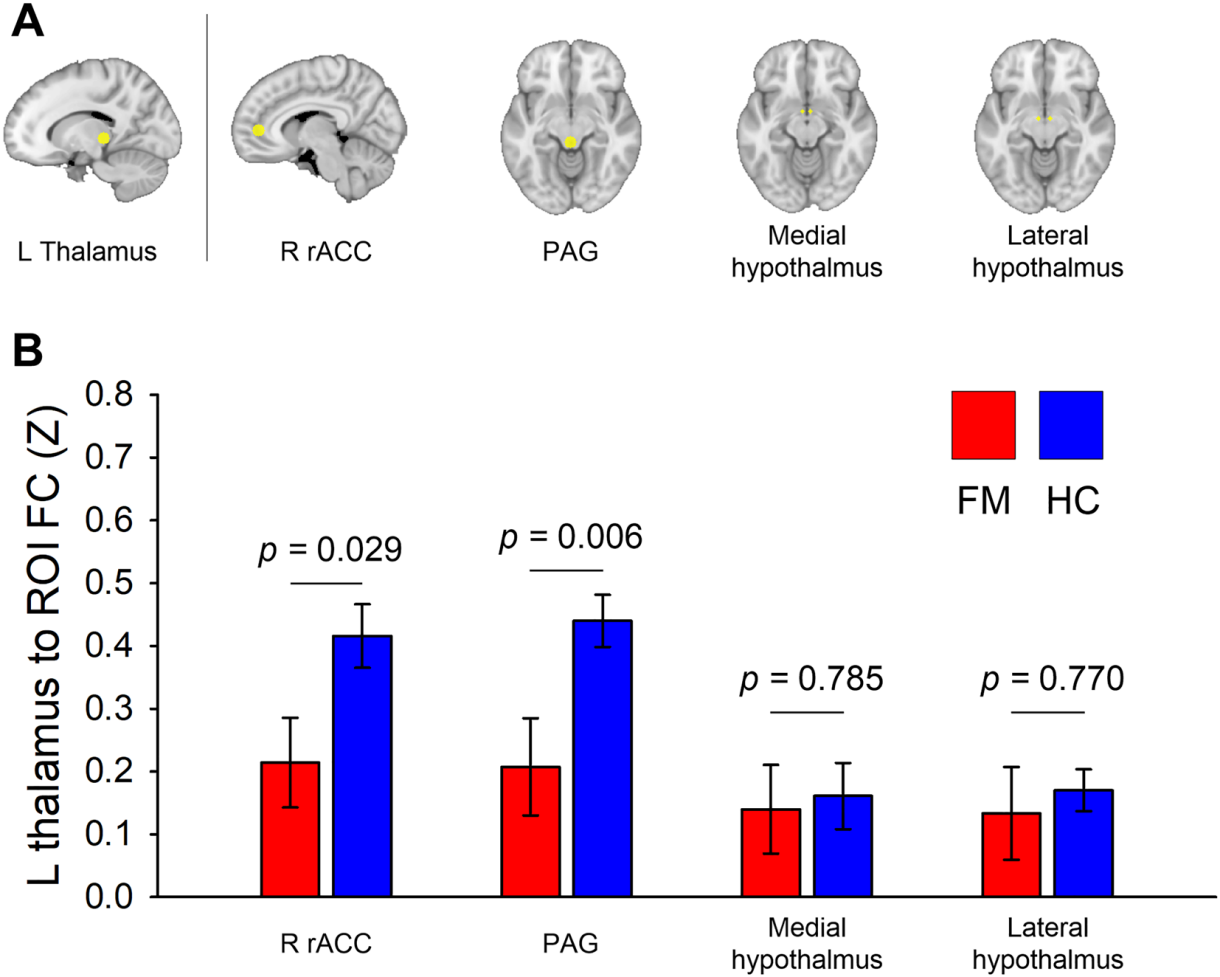
Thalamus functional connectivity analysis using ROI. (**A**) Spherical ROIs with the 6-mm radius were defined for right rACC (x = 8, y = 46, z = 4)^19^ and PAG (x = 0, y =  − 32, z =  − 10)^43,44^. Spherical ROIs with the 2-mm radius were defined for bilateral medial (x =  ± 4, y =  − 2, z =  − 12) and lateral hypothalamus (x =  ± 6, y =  − 2, z =  − 12)^45^. (**B**) Group differences in thalamus to ROI functional connectivity between people with FM and HCs. Bar graphs were expressed as mean ± standard error of the mean. rACC, rostral anterior cingulate cortex; PAG, periaqueductal gray; ROI, region of interest.

ROI based functional connectivity analysis demonstrated significantly reduced connectivity between thalamus and PAG (*f*(1, 29) = 8.876, *p* = 0.006, *q* = 0.024) and marginally reduced connectivity thalamus and rACC (*f*(1, 29) = 5.256, *p* = 0.029, *q* = 0.058) in the FM group compared to HC group after multiple comparison correction. There were no significant group difference in thalamus-medial hypothalamus (*f*(1, 29) = 0.076, *p* = 0.785, *q* = 0.785) and thalamus-lateral hypothalamus (*f*(1, 29) = 0.087, *p* = 0.770, *q* = 0.785) connectivity (Fig. 5B).

We did not find any significant correlation between depressive symptoms and brain measures (*p* > 0.05) (Supplementary Table S1).

## Discussion

We demonstrated structurally specific thalamic alteration and its dysfunctional interaction with the cortex in individuals with FM by using multiple and complementary approaches, including volumetric, vertex-based subcortical shape, cortical thickness, structural and functional connectivity analyses. The study results confirmed our hypothesis that individuals with FM exhibited focal atrophy of the left posterior thalamus, including the pulvinar and VPL nuclei. The atrophied thalamic displacement (inward) was negatively associated with the left IPL thickness, which was opposite to what was found in HCs. In addition, individuals with FM showed abnormal functional connectivity between the left posterior thalamus and IPL (including angular gyrus) and, further, associated with lower pain threshold measured by electrical nociceptive stimuli.

Vertex-wise subcortical shape analysis, not a total volume measurement, appeared to better detect localized and pathological changes in individuals with FM. We found an inward deformation of the posterior thalamus, approximately correspondent to the pulvinar and VPL regions with a peak voxel located in the pulvinar nuclei. Although different analytical methods were applied, structural or functional thalamic abnormalities seemed to converge into the posterior aspect of the thalamus in studies comparing individuals with FM to controls. For example, a regional decrease of the posterior thalamic volume and white matter integrity was found in individuals with FM using voxel-based morphometry and diffusion tensor imaging, respectively^30,48^_._ Moreover, individuals with FM showed hypoactivity of the left pulvinar nuclei in response to low stimulus pressures, which induced the same pain intensity as HCs^19^.

It remained unanswered about the mechanism that drives pathological changes of the thalamus or whether this precedes or changes with a disease process. Given the role of the posterior thalamus (VPL/pulvinar) nuclei in relaying nociception from the spinal cord to the thalamus^49^, prolonged nociception, which might cause gray matter atrophy^50^ through spinothalamic projections, would be a reasonable explanation^51^. This study, however, could not rule out the alternative explanation. Notably, it was shown that pulvinar lesion led to central pain, and pulvinotomy reduced intractable malignant pain^49,51^, highlighting the pulvinar involvement in the emergence of chronic pain symptoms.

We found out that the HC group exhibited positive structural coupling between the left posterior thalamus and the left inferior parietal and right cuneus areas. In contrast, these patterns were largely absent or inversed in the IPL regions in the FM group. In other words, FM individuals with more inward thalamic deformation showed a thicker cortex. The SC network has been suggested to reflect mutually trophic reinforcement^52^, genetic influence^53,54^, neurodevelopment and maturation^55^, neurodegeneration^56^, and experience-related plasticity^57^. In light of the findings that pulvinar nuclei are connected with the posterior parietal and the occipital cortex, as demonstrated by diffusion tractography and functional connectivity studies^31–33^, we postulated that these covaried changes in these regions might indicate chronic nociception-induced shared plasticity in anatomically and functionally related regions. Noteworthy, the pulvinar nuclei, higher-order nuclei of the thalamus, appeared to play a regulatory role in information transmission across cortical areas based on attentional allocation beyond the simple relaying of the pain signal as previously thought^27,58^. Thus, we speculated that alterations in the pulvinar structure or function might have consequences in connected cortical regions.

The superior frontal cortex, which was thinner in the FM group, is not classically involved in nociceptive processing^59^, but more implicated in pain modulatory role. A prior study by Jensen et al. (2013) also found a similar result with a thinner left superior frontal cortex, which appeared progressive depending on chronicity^60^. However, in our present study, the thinner superior frontal cortex was associated with a lower pain threshold of the thenar muscle, a pain-free body site, but not with duration or other clinical variables. Since individuals with FM showed significantly low pain threshold than controls, this result supports the notion that the impaired pain modulatory role of the central nervous system contributes to augmented pain in FM^61^. We did not find any significant SC pattern between the posterior thalamus and the superior frontal gyrus. We reasoned that it could be attributed to the regional specificity of the posterior thalamus of our results and its preferential connectivity with the parietal or occipital cortices.

In line with the SC results, the functional connectivity analysis showed enhanced connectivity between the posterior thalamus and IPL in individuals with FM. Recent evidence suggested that the functional network between the ventral pulvinar nuclei and interconnected cortex constitutes an attentional network^62^. Further, the angular gyrus has been implicated in the top-down attentional control of pain^63,64^. The relevant example of this claim is that increased low-frequency oscillations of the left angular gyrus were correlated to music-induced analgesia in individuals with FM^65^. The attentional network has a crucial role in selecting information to be attended through competing sensory inputs^66^. Accordingly, we hypothesized that the abnormal relationship between the pulvinar nuclei and the angular gyrus might negatively impact a neural decision regarding which information is more salient and thus which has to be focused. Therefore, this abnormal interaction might explain a hypersensitivity to both painful and non-painful stimuli of individuals with FM^10,12,13^.

Furthermore, our results add to the evidence supporting the hyperconnectivity of the posterior thalamic nuclei contributing to abnormal pain experience in individuals with FM by showing a negative association with the posterior thalamus-IPL functional connectivity and electrical pain threshold on the hand dorsum, which was significantly lower for individuals with FM compared with controls. This result is partly in line with a recent study examining TC connectivity dynamics in migraine, which found an abnormal posterior thalamus dynamic functional connectivity with the primary somatosensory cortex^26^ or visual cortex^67^, further associated with the headache frequency of migraine. In a later study applying similar methods, the authors found higher dynamic connectivity between ventral lateral/VPL and primary somatosensory cortex in individuals with chronic back pain^68^.

A potential mechanism of such hyperconnectivity might be related to reduced gamma-aminobutyric acid (GABA), an inhibitory neurotransmitter, of the thalamus. In the previous study with neuropathic pain, Henderson et al. (2013) found an association between the decrease in GABA level of the ventroposterior thalamus and greater ventroposterior thalamus and somatosensory cortex connectivity^69^. They proposed that the flawed GABAergic input to the ventroposterior thalamus leads to TC dysrhythmia and eventually strengthens TC connectivity^69^. There are, however, no reports demonstrating decreased thalamic GABA concentration in individuals with FM; this is partly because of a scarcity of studies examining the neurotransmitter system of FM. Since an imbalance between inhibitory and excitatory neurotransmission is strongly implicated in augmented pain processing in FM^15,16,70^, that hypothesis would be testable.

In an additional ROI-to-ROI functional connectivity analysis, we observed less posterior thalamic connectivity with the PAG, known to exert descending pain modulation^71^. This hypoconnectivity agrees with previous findings of decreased functional connectivity with the PAG in FM^20,72^, which supports the idea of common mechanisms involved in inefficient endogenous pain modulation in chronic primary pain disorders^73^. Interestingly, Cummiford et al. (2016) reported that higher functional connectivity between the left ventrolateral thalamus and PAG at baseline predicted a greater analgesic effect in FM after active transcranial direct current stimulation over the primary motor cortex treatment^28^. We speculated based on that study that higher thalamo-PAG connectivity might function as a reservoir for endogenous pain modulation in FM. Additionally, it was found that distraction from the noxious heat reduced pain perception but increased covariation of the BOLD signal between posterior thalamus and PAG . However, this increased functional interaction was not found during painful stimulation only^74^. We further speculated that the reduced posterior thalamus-PAG connectivity might underlie a failure in attentional pain modulation and partly explain reduced attention or cognitive function widely shown in FM^75^.

Some study limitations should be considered in interpreting the results. First, even though FM participants had 3 days of drug washout before the experiment, it is still insufficient to eliminate cumulative medication effect on the brain. Second, we cannot test a direct association between the TC-SC strength and pain threshold in FM since the SC analysis did not produce an individual value. However, it would be resolved by our complementary functional connectivity analysis, which demonstrated a comparable result supporting alteration in the posterior thalamo-IPL network and its association with pain threshold. Third, our studies relied on a small sample size which is prone to low reproducibility^76^. Therefore, the current results need to be validated in a sufficient sample size for more reliability. Along with this, considering the significant effect of sex in brain structure and function in chronic pain disorders, including FM^77^, our results may not generalize to FM population. Future approaches to systemically assess similarity or difference of sex effect on neural substrates of FM will help overcome this limitation.

Methodologically, more advanced approaches such as dynamic functional connectivity^26,67,68^ or systematic assessment of each thalamic nuclei and their connectivity will shed light on understanding the neural correlates subserving the complex nature of this pain disorder.

In conclusion, our findings provide evidence of regional atrophy of the posterior thalamus (pulvinar/VPL) and disrupted structural and functional networks with inferior parietal regions in individuals with FM. Given the posterior thalamo-IPL network involvement in pain modulation and attentional process, the current findings suggest that the abnormal structural and functional posterior thalamo-IPL network might influence pain sensitivity, potentially linked to elevated pain level in FM.

## Supplementary Information

Supplementary Information.
