# Supplementary material for: Structural and functional thalamocortical connectivity study in female fibromyalgia
Source: Sci Rep. 2021 Dec 2;11:23323. doi: 10.1038/s41598-021-02616-1 (PMC8640058; doi:10.1038/s41598-021-02616-1)
Supplement: Supplementary file 1 — Supplementary Information. [file 41598_2021_2616_MOESM1_ESM.docx]

**Supplementary Table S1.** Correlation between brain measures and depressive symptoms measured by Beck Depression Inventory.

|  | | FM (n = 19) | | HC (n = 20) | |
| --- | --- | --- | --- | --- | --- |
| Brain measures | Brain regions | *r* | *p* | *r* | *p* |
| Cortical thickness | Left superior frontal cortex | -0.16 | 0.52 | 0.05 | 0.84 |
| Thalamus shape | Left posterior thalamus | -0.16 | 0.52 | 0.08 | 0.74 |
| Functional connectivity with left posterior thalamus* | Left inferior parietal lobule | 0.15 | 0.64 | -0.08 | 0.73 |
|  | Right inferior parietal lobule | 0.26 | 0.41 | -0.16 | 0.49 |
|  | Left middle/inferior temporal gyrus | -0.30 | 0.34 | 0.31 | 0.19 |
|  | Periaqueductal gray | 0.30 | 0.35 | -0.24 | 0.32 |

*n = 12 for FM. FM, fibromyalgia; HC, healthy control.
